# Oral Sodium Chloride in the Prevention of Contrast-Associated Acute Kidney Injury in Elderly Outpatients: The PNIC-Na Randomized Non-Inferiority Trial

**DOI:** 10.3390/jcm12082965

**Published:** 2023-04-19

**Authors:** Cecilia Suárez Carantoña, Carlos Escobar Cervantes, Martín Fabregate, Mónica López Rodríguez, Nuria Bara Ledesma, Javier Soto Pérez-Olivares, Raúl Antonio Ruiz Ortega, Genoveva López Castellanos, Andreina Olavarría Delgado, Javier Blázquez Sánchez, Vicente Gómez del Olmo, Myriam Moralejo Martín, María Belén Pumares Álvarez, María de la Concepción Sánchez Gallego, Pau Llàcer, Fernando Liaño, Luis Manzano

**Affiliations:** 1 Internal Medicine Department, Hospital Universitario Ramón y Cajal, IRYCIS, CTRA M-607 Colmenar Viejo, Km 9.10, 28034 Madrid, Spain; 2 Faculty of Medicine and Health Sciences, Universidad de Alcalá (UAH), Pl. de San Diego, s/n, 28801 Alcalá de Henares, Spain; 3 Cardiology Department, Hospital Universitario La Paz, IdiPaz, P.º de la Castellana, 261, 28046 Madrid, Spain; 4 Centro de Innovación en Tecnología para el Desarrollo Humano, Universidad Politécnica de Madrid (itdUPM), Av. Complutense s/n, 28040 Madrid, Spain; 5 Radiology Department, Hospital Universitario Ramón y Cajal, IRYCIS, CTRA M-607 Colmenar Viejo, Km 9.10, 28034 Madrid, Spain; 6 Department of Nephrology, Hospital Universitario Ramón y Cajal, IRYCIS, CTRA M-607 Colmenar Viejo, Km 9.10, 28034 Madrid, Spain

**Keywords:** contrast-associated acute kidney injury, contrast-enhanced computed tomography, elderly

## Abstract

Objective: We aimed to test the non-inferiority of oral versus intravenous hydration in the incidence of contrast-associated acute kidney injury (CA-AKI) in elderly outpatients undergoing a contrast-enhanced computed tomography (CE-CT) scan. Methods: PNIC-Na (NCT03476460) is a phase-2, single-center, randomized, open-label, non-inferiority trial. We included outpatients undergoing a CE-CT scan, >65 years having at least one risk factor for CA-AKI, such as diabetes, heart failure, or an estimated glomerular filtration rate (eGFR) of 30–59 mL/min/1.73 m². Participants were randomized (1:1) to oral sodium-chloride capsules or intravenous hydration. The primary outcome was an increase in serum creatinine >0.3 mg/dL or a reduction in eGFR >25% within 48 h. The non-inferiority margin was set at 5%. Results: A total of 271 subjects (mean age 74 years, 66% male) were randomized, and 252 were considered for the main analysis (per-protocol). A total of 123 received oral hydration and 129 intravenous. CA-AKI occurred in 9 (3.6%) of 252 patients and 5/123 (4.1%) in the oral-hydration group vs. 4/129 (3.1%) in the intravenous-hydration group. The absolute difference between the groups was 1.0% (95% CI −4.8% to 7.0%), and the upper limit of the 95% CI exceeded the pre-established non-inferiority margin. No major safety concerns were observed. Conclusion: The incidence of CA-AKI was lower than expected. Although both regimens showed similar incidences of CA-AKI, the non-inferiority was not shown.

## 1. Introduction

The use of the contrast-enhanced computed tomography (CE-CT) scan has been steadily increasing in recent years for improving diagnostic accuracy. Iodinated contrast medium may have a negative impact on renal function in patients with and without chronic kidney disease (CKD) [1]. Contrast-associated acute kidney injury (CA-AKI), formerly known as post-contrast acute kidney injury, is a sudden decrease in renal function within 48 h following the intravascular administration of iodinated contrast medium, regardless of the cause [2].

The incidence of CA-AKI in the overall population undergoing a CE-CT scan is generally low, but may increase when some conditions are present, such as aging, diabetes, or previous renal impairment [3,4]. CA-AKI is associated with renal failure, longer hospital stays, and mortality [5]. Although the mechanism of the injury is not fully clarified, increased plasma viscosity, tubuloglomerular feedback response, local renal hypoxia, tubular cell toxicity, or oxidative stress have been involved in the pathophysiology of CA-AKI [1,6,7].

The prevention of CA-AKI among patients at risk undergoing a CE-CT scan is currently recommended [8]. Even though different strategies have been proposed [9], only previous hydration has shown to effectively prevent CA-AKI [10]. Intravenous sodium chloride (NaCl) hydration has been extensively studied, mainly in patients undergoing percutaneous coronary intervention, particularly in the acute setting [11,12] However, oral volume expansion could be a more suitable option in some cases. In fact, from 2019, the National Institute for Health and Care Excellence Guideline for acute kidney injury encourages oral hydration before and after procedures using intravenous iodine-based contrast media in adults at an increased risk of CA-AKI [13]. Unfortunately, large studies have not yet been performed on CA-AKI prevention with oral hydration, and those that include oral hydration are scarce and have a limited statistical power, leading to heterogeneous results [14,15,16]. Regarding oral NaCl hydration, a previous trial [17], which included patients with CKD III undergoing various contrast-enhanced radiological procedures, had suggested that oral NaCl hydration was as efficient as intravenous saline hydration in the prevention of CA-AKI.

We hypothesized that oral hydration with NaCl is non-inferior to intravenous hydration in elderly outpatients at risk of CA-AKI undergoing a CE-CT scan. The main objective of this study was to test the non-inferiority of oral compared to intravenous NaCl hydration on the incidence of CA-AKI following a CE-CT scan. The secondary aims included comparing the continuous efficacy assessments between both arms and describing the safety profile within the 48 h following the CE-CT scan.

## 2. Materials and Methods

### 2.1. Study Design

PNIC-Na was a prospective, phase-2, single-center, randomized, parallel-group, open-label, non-inferiority clinical trial, registered at ClinicalTrials.gov (NCT03476460).

### 2.2. Participants

Outpatients undergoing a CE-CT scan, older than 65 years having at least one of the following risk factors: diabetes mellitus, stable heart failure, or CKD (estimated glomerular filtration rate, eGFR [MDRD-4] between 30 and 59 mL/min/1.73 m^2^) were included. We excluded patients with eGFR <30 mL/min/1.73 m^2^, hypokalemia, receiving intravenous iodinated contrast within 15 days prior to screening, nephrotoxic drugs in the previous 72 h or immediately after the procedure, decompensated chronic disease, hypersensitivity to contrast, hyperchloremia, or hypernatremia. Study discontinuation criteria included voluntary withdrawal of the participant or safety reasons. The trial was approved by the Research Ethics Committee of Hospital Universitario Ramón y Cajal (protocol ref.: 124-13) and the regulatory authorities and conducted in accordance with the trial protocol. Participants were recruited at Hospital Universitario Ramón y Cajal, Madrid, Spain, between April 2014 and November 2019. All participants gave signed informed consent before being enrolled in the study. The trial was independently monitored by the Unit of Clinical Research and Clinical Trials, ISCIII National Network.

### 2.3. Randomization and Masking

Subjects were randomly assigned (1:1) to receive either oral or intravenous hydration. The Clinical Biostatistics Unit of our institution built a computer-generated randomization sequence by blocks of size six to guarantee the balance. An automated allocation system was used, so that the assignment sequence was unknown to the investigators. Treatment allocation was performed by opening numbered, sealed, and opaque envelopes. Once randomized, the Pharmacy Department supplied the study treatment.

### 2.4. Procedures

Subjects in the oral hydration arm were scheduled to take NaCl capsules (500 mg) and free water intake every eight hours within the 48 h prior to contrast exposure and an additional dose 12 h later. The NaCl dose in the oral arm was calculated according to subject body weight (100 mg/kg) to be equivalent to that in the intravenous hydration arm. Intravenous hydration consisted in the administration of 11 mL/kg of 0.9% NaCl, according to current recommendations [8]. The administration was performed at the hospital during the hour before CE-CT scan (3 mL/kg/h) and through the four hours later (2 mL/kg/h). The contrast used for the CT scan was Iodixanol (320 mg iodine/mL), with an approximate volume of 100 mL, and an infusion rate of 2–5 mL/s. Metformin was stopped from 48 h before to 24 h after the CE-CT scan. Water and salt intake was not limited.

At inclusion, demographic data, physical examinations, comorbidities, laboratory parameters, and concomitant treatments were recorded. The compliance of NaCl capsules was registered for participants in the oral arm. Physical examination, collection of blood and urine samples, and adverse event recording were performed immediately before and 24 and 48 h after contrast administration. If the patient was unable to attend the site for follow-up visits, study nurses went to the participant’s home. Safety was monitored by the recording of adverse events, including severity and relationship with study medication. An adverse event was considered as serious if it resulted in death, threatened the life of the subject, required hospitalization, or caused permanent or significant disability or incapacity.

### 2.5. Outcomes

Efficacy assessments were performed immediately prior to contrast administration (baseline) and then 24 and 48 h later. The main efficacy outcome for CA-AKI was defined as an increase in serum creatinine >0.3 mg/dL or a reduction >25% in eGFR [MDRD-4], within 48 h from baseline. Additionally, changes in efficacy laboratory assessments (eGFR, serum creatinine, cystatin C, albumin-to-creatinine ratio, and electrolytes) were analyzed. Adverse events were registered, including severity and relationship with study medication.

### 2.6. Statistical Analysis

Sample size was calculated to assess the non-inferiority of oral compared to intravenous hydration. We expected a primary outcome rate of 7% in the intravenous arm, based on a conservative estimation according to the available evidence at the time of study design for elderly patients with diabetes, heart failure, or CKD [18]. We considered a priori a difference of no more than 5% in the incidence of CA-AKI in the oral compared to the intravenous arm (non-inferiority margin) to be acceptable. Thus, 266 participants, 133 per arm, were required to ensure at least 80% power at a significance level of α = 2.5% (one-sided).

Variables are presented as mean ± standard deviations or median (interquartile ranges) or frequencies and percentages. The absolute difference in primary outcome rates (%) between both arms was calculated with the 95% confidence interval (CI) using the Wilson’s method. Non-inferiority was considered if the upper limit of the two-sided 95% CI of the absolute difference in primary outcome rates was <5%. The main analysis was performed in the per-protocol (PP) population. For sensitivity analysis, the main analysis was carried out on the intention-to-treat (ITT) population and then repeated with worst-case and best-case imputations for missing outcome data. In addition, we reassessed the non-inferiority considering as main outcome the KDIGO definition of AKI: an increase in serum creatinine ≥ 0.3 mg/dL or 1.5 times baseline, within 48 h after iodinated contrast [19]. We performed subgroup analyses for the risk factors defined as inclusion criteria (e.g., elderly, diabetes, heart failure, and chronic kidney disease) to assess whether the main analysis was consistent across post hoc subgroups. For the safety analysis, we considered all the patients who received at least one dose of study drug.

All other group comparisons were of exploratory nature. We used the Pearson’s chi-square and Fisher’s exact tests and the independent sample Student’s t or Mann–Whitney’s tests, as appropriate. All available data were included, with no imputation of missing values or adjustments for multiplicity of contrasts. For the primary outcome, a significance level of 0.025 (one-sided) was considered. For all other comparisons, we considered two-sided *p*-values of 0.05. IBM-SPSS Statistics v24.0 and R v4.1.2 were used.

## 3. Results

**Figure 1:**
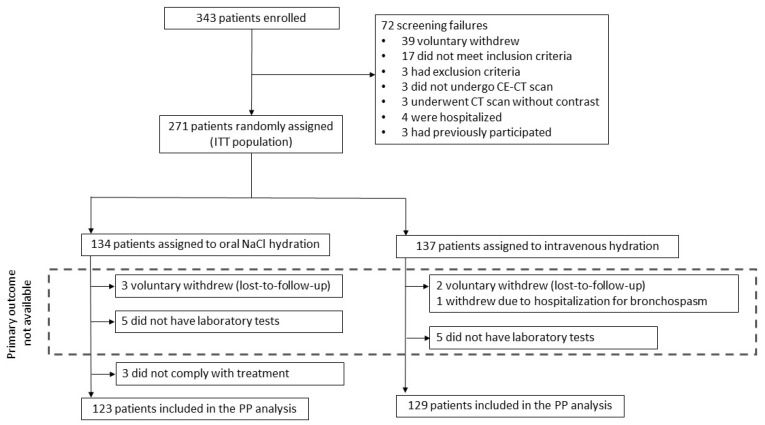
Trial profile. CE-CT: contrast-enhanced computerized tomography; ITT: intention-to-treat; PP: per-protocol.

A total of 343 outpatients were assessed for eligibility. After excluding 72 subjects (Figure 1), 271 were included in the ITT and safety analyses, of whom 134 were randomly assigned to oral hydration and 137 to intravenous hydration. Finally, 19 (7.0%) did not meet the criteria for PP analysis. Therefore, the PP population consisted of 252 patients, of whom 123 received oral hydration and 129 intravenous hydration. The study ended once the enrollment goal was reached and the follow-up period for all participants was completed.

The mean age was 74.4 ± 6.4 years; 180 (66.4%) of 271 randomized participants were men; 112 (41.3%) had CKD; 198 (73.1%) were diabetics; and 46 (17.0%) had heart failure. Clinical characteristics and concomitant treatments at inclusion were well balanced, and no clinically relevant differences were found between the oral and intravenous groups, both in the PP and ITT populations (Table 1 and Table A1). The mean volume contrast material administered was 99.6 ± 1.9 mL (range 90.0–117.0 mL).

**Figure 2:**
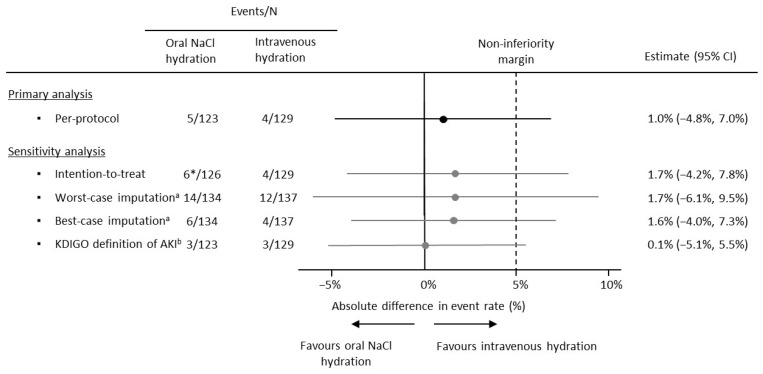
Forest plot of the main outcomes (primary and sensitivity analyses). The results are given as absolute risk differences (black points) with a 95% confidence interval (black line) for the primary outcome (i.e., decrease in eGFR [MDRD-4] > 25% or increase in serum creatinine > 0.3 mg/dL, within 48h after CE-CT scan). The non-inferiority of oral hydration would be shown if the upper limit of the 95% CI of the absolute risk difference between the groups was less than 5% (non-inferiority margin, indicated by the black dashed line). The 95% confidence interval for the difference between the two independent proportions was calculated according to Wilson’s method. * A primary event took place in a subject excluded from the per-protocol analysis due to a lack of adherence to the oral treatment. ^a^ Sensitivity analyses were performed on the intention-to-treat population, with missing outcome data imputed as if all (worst case) or none (best case) of them had a primary outcome. ^b^ Non-inferiority analysis was repeated, considering as the main outcome the definition of acute kidney injury (AKI) proposed by the Acute Kidney Injury Working Group of KDIGO (Kidney Disease: Improving Global Outcomes), meaning an increase in serum creatinine ≥ 0.3 mg/dL or 1.5 times the baseline, within 48 h after iodinated contrast [19].

The primary outcome (decrease in eGFR >25% or increase in serum creatinine >0.3 mg/dL, within 48 h after CE-CT scan) occurred in 9 (3.6%) of 252 patients in the PP population: 5/123 (4.1%) in the oral-hydration group vs. 4/129 (3.1%) in the intravenous-hydration group. The absolute difference in CA-AKI incidence rates was 1.0% (95% CI −4.8% to 7.0%). As the upper limit of the 95% CI of the absolute rate difference (7.0%) was higher than the non-inferiority margin (5.0%), non-inferiority was not met (Figure 2). In the ITT population, there was one additional primary event in the oral-hydration group, which was excluded from the PP analysis due to a lack of adherence to the oral treatment. The primary outcome was missing in 16/271 (5.9%) patients in the ITT population. Both ITT and sensitivity analyses (worst-case and best-case imputation and KDIGO definition of AKI as the main outcome) showed point estimates and 95% CIs similar to those of the PP analysis (Figure 2). As for the subgroup analyses, the difference in risk of CA-AKI between the oral and intravenous hydration exceeded the non-inferiority margin across all the subgroups (Table A2).

Regarding post-contrast differences in continuous efficacy assessments within the 48-h follow-up period in the per-protocol population (Table 2), serum creatinine values were higher in the oral-hydration group at 24 h 1.10 ± 0.33 vs. 1.02 ± 0.29 mg/dL; *p* = 0.042. However, at 48 h, serum creatinine was not significantly different between both groups. Meanwhile, eGFR, urea, cystatin C, albumin-to-creatinine ratio, and serum sodium and potassium levels did not change between oral and intravenous hydration within the 48-h follow-up period. Similar results were found when we repeated this analysis within the ITT population (Table A3).

A description of the adverse events reported in the ITT population are presented in Table 3. There were 31 adverse events in the oral-hydration group, and 27 were considered related to the study treatment at the investigators’ discretion, and none of them were a serious adverse event. Gastrointestinal (GI) symptoms, including nausea, vomiting, and abdominal discomfort, were the most common adverse events in the oral arm (26/134, 19.4%). No significant association between GI symptoms and incidence of CA-AKI was found in the oral arm (5.3% for GI vs. 3.8% for non-GI; *p* = 0.574). In the intravenous-hydration group, six adverse events were reported, and two of them related to study treatment. Two patients receiving intravenous hydration presented serious adverse events (bronchospasm and infection); none of them related to the study treatment.

## 4. Discussion

In this clinical (PNIC-Na) trial in elderly outpatients at risk of CA-AKI undergoing a CE-CT scan, the non-inferiority of oral compared to intravenous hydration was not established. It should be emphasized that the incidence of CA-AKI was much lower than expected (4.1% and 3.1% in the oral and intravenous arms, respectively). No major safety concerns were found. In the oral regimen, the most common adverse events were mild gastrointestinal symptoms, not associated with a higher incidence of CA-AKI.

It is worth noting that we chose CA-AKI as the main outcome because in clinical practice it is not always possible to determine the etiology of renal deterioration after contrast medium administration. Furthermore, we considered that the focus should be on reducing the incidence of AKI regardless of the cause. In this regard, the definitions used were heterogeneous, using causal or temporal association criteria, which hinder the comparison and interpretation of existing evidence. To date, few studies have been carried out to assess renal protection with oral hydration in outpatients at risk of CA-AKI [14,15,16]. In particular, at the time our study was conceived, a previous trial [17] with sodium chloride suggested that oral saline hydration might not be inferior to intravenous saline hydration in the prevention of CA-AKI in patients with stage 3 renal disease. Likewise, in our study, the dose of oral NaCl was similar to that used in the previous trial (100 mg/kg subject body weight) [17], in both cases combined with free water intake, even though the previous evidence supporting oral hydration was limited by small patient numbers. In this context, the PNIC-Na trial was designed to compare oral vs. intravenous NaCl hydration strategies in elderly outpatients at risk for CA-AKI after a CE-CT scan.

Our study shows a slight difference in the CA-AKI incidence rates between the oral and intravenous arms (1.0%). However, as the upper limit of the 95% CI (7.0%) exceeded the predefined margin of 5%, the non-inferiority of oral compared with intravenous NaCl hydration was not shown. The primary outcome was analyzed in the PP population, as this is a better approach in non-inferiority clinical trials [20]. Nevertheless, our findings were confirmed in the sensitivity analyses, providing evidence in favor of the robustness of our key finding.

The risk of developing CA-AKI in patients who receive iodinated contrast has probably been overestimated. Two meta-analysis including around 19,000 patients who had received intravenous contrast media showed CA-AKI incidences of 5.0% and 6.4% [21,22], significantly lower than previously thought. Furthermore, a recent study has reported an incidence of CA-AKI as low as 0.9% among patients with moderate CKD after a CE-CT scan [3]. Current procedures using minimum volume, non-ionic contrast material might explain why the CA-AKI incidence is low, even in high-risk patients [10,23]. In fact, in the current trial (PNIC-Na), only 3.6% of the overall study population developed CA-AKI.

It should be considered that, in contrast to other studies, all participants in the PNIC-Na trial were aged over 65 years, with at least an additional risk factor, but patients with an eGFR lower than 30 mL/min/1.73 m² were not included out of safety considerations. Most of our patients had hypertension and diabetes, nearly half of them CKD, and one out of six heart failure. Therefore, the study was conducted in a selected elderly population at risk of CA-AKI, providing relevant information on this population, which was not targeted by previous studies [15].

Although our trial did not show the non-inferiority of oral NaCl compared to intravenous saline in the prevention of CA-AKI, previous studies had shown that oral hydration was not associated with more risk of CA-AKI in patients undergoing a CE-CT scan [15,24,25]. In particular, the recent NICIR study showed the non-inferiority of oral compared to intravenous hydration in the prevention of CA-AKI in patients with stage IIIb CKD referred for an elective CE-CT scan [25]. In this population, the CA-AKI rate was 4.4% in the oral and 5.3% in the intravenous hydration arm. These rates were slightly higher than the ones observed in our study (4.1% and 3.1%, respectively), probably due to differences in baseline renal function between both populations. Concerning the subgroups of particular interest, it is noteworthy that the overall CA-AKI incidence in very elderly patients (≥80 years) was more than four times that of younger patients. Current clinical guidelines recommend intravenous hydration in patients with an eGFR < 30 mL/min/1.73 m^2^, regardless of age [8,26]. Thus, despite the limited number of events in our study, given its potential interest and the low risk of this prophylaxis, we suggest that further research could be developed in this line.

We also provided further insight into the change over time in efficacy assessments for the oral and intravenous NaCl hydration arms. Thus, only serum creatinine was slightly higher in the oral arm at 24 h, but it is not clinically relevant and might be related to minor differences in baseline values between both groups. Likewise, meaningless differences over the time were observed in other renal function biomarkers. Our results are consistent with a recent study that did not find a negative impact of CE-CT scan in an emergency setting on long-term kidney function [27]. As a result, although some renal impairment can occur after contrast administration, this is usually small and transitory.

Our findings should be interpreted considering some limitations. First, the incidence of CA-AKI was much lower than expected at the time of study design. A limited number of events in the sample may have impacted the failure to demonstrate the non-inferiority of oral hydration. Thus, a larger sample size may have narrowed the 95% CI. Moreover, the definition of CA-AKI considered in our study is slightly different from that proposed in the clinical guidelines for AKI [19], which could have discreetly modified the number of main outcomes. However, a sensitivity analysis was carried out using that definition, also failing to demonstrate the non-inferiority. Serial measurements of serum and urinary osmolality may have been informative about the effect of the oral intervention on plasma volemia and osmolarity. Although they were not available in our trial, we assessed serum and urinary sodium throughout the study. Lastly, the PNIC-Na was a single-center study, which might limit its external validity.

## 5. Conclusions

In summary, the PNIC-Na trial showed, in a wide sample of elderly patients with relevant comorbidities, that the incidence of post-contrast acute kidney injury after a CE-CT scan was much lower than expected in both arms. However, we could not establish the non-inferiority of the oral NaCl compared to intravenous hydration in the prevention of CA-AKI.

## Figures and Tables

**Table 1:** Patient characteristics at inclusion in the study (per-protocol population).

	Per-Protocol Population	
	Oral NaCl Hydration (n = 123)	Intravenous Hydration (n = 129)
**Demographic data**		
Age, years	74.1 ± 6.1	74.6 ± 6.8
Sex, male	89 (72.4%)	79 (61.2%)
**Comorbidities**		
Hypertension	94 (76.4%)	98 (76.0%)
Diabetes	87 (70.7%)	97 (75.2%)
Smoking		
Ex-smoker	74 (60.2%)	66 (51.2%)
Current smoker	5 (4.1%)	9 (7.0%)
Cancer	68 (55.3%)	59 (45.7%)
Chronic kidney disease ^a^	53 (43.1%)	47 (36.4%)
Heart failure	16 (13.0%)	25 (19.4%)
Peripheral artery disease	11 (8.9%)	8 (6.2%)
**Laboratory parameters**		
eGFR, mL/min/1.73 m^2^	66.6 ± 19.4	69.0 ± 19.6
Serum creatinine, mg/dL	1.09 ± 0.31	1.02 ± 0.29
Cystatin C, mg/dL	1.36 ± 0.39	1.41 ± 0.46
Albumin-to-creatinine ratio, mg/g	17.4 [71.8]	19.4 [52.7]
Urea, mg/dL	49.1 ± 16.2	46.7 ± 18.8
Serum sodium, mg/dL	139.2 ± 2.6	139.6 ± 3.1
Serum potassium, mg/dL	4.5 ± 0.4	4.5 ± 0.5
HbA1c, %	6.6 ± 1.0	6.7 ± 1.5
BNP, pg/mg	56.6 [129.1]	54.0 [70.7]
**Concomitant medication**		
Non-diuretic antihypertensives	95 (77.2%)	97 (75.2%)
Non-insulin antidiabetic drugs	81 (65.9%)	86 (66.7%)
Lipid-lowering agents	76 (61.8%)	79 (61.2%)
Diuretics	48 (39.0%)	45 (34.9%)

Patient characteristics collected at inclusion, before the contrast-enhanced CT scan, prior to study treatment exposure. Data are expressed as n (%), mean ± standard deviation, or median [interquartile range]. ^a^ Chronic kidney disease defined as eGFR (MDRD-4) < 60 mL/min/1.73 m^2^. Abbreviations: eGFR: estimated glomerular filtration rate according to MDRD-4; BNP: brain natriuretic peptide.

**Table 2:** Differences in continuous efficacy assessments between oral- and intravenous-hydration groups within the 48-h follow-up period in the per-protocol population.

	Per-Protocol Population		
	Oral NaCl Hydration (n = 123)	Intravenous Hydration (n = 129)	*p*
**eGFR, mL/min/1.73 m^2^**			
24 h	66.4 ± 18.9	68.8 ± 18.9	0.299
48 h	66.1 ± 19.7	67.9 ± 19.6	0.477
**Serum creatinine, mg/dL**			
24 h	1.10 ± 0.33	1.02 ± 0.29	0.042
48 h	1.10 ± 0.36	1.04 ± 0.31	0.121
**Cystatin C, mg/dL**			
24 h	1.38 ± 0.44	1.33 ± 0.46	0.418
48 h	1.36 ± 0.43	1.36 ± 0.46	0.901
**Albumin-to-creatinine ratio, mg/g**			
24 h	17.2 [63.7]	17.0 [56.2]	0.720
48 h	17.6 [60.2]	18.2 [41.0]	0.688
**Urea, mg/dL**			
24 h	43.7 ± 18.7	42.2 ± 18.2	0.535
48 h	46.0 ± 19.5	43.5 ± 20.1	0.338
**Serum sodium, mg/dL**			
24 h	139.4 ± 2.6	139.3 ± 2.6	0.764
48 h	139.2 ± 2.7	139.4 ± 2.6	0.458
**Serum potassium, mg/dL**			
24 h	4.4 ± 0.4	4.4 ± 0.5	0.893
48 h	4.4 ± 0.4	4.5 ± 0.5	0.645

Data expressed as mean ± standard deviation or median [interquartile range]. eGFR: estimated glomerular filtration rate according to MDRD-4.

**Table 3:** Safety analysis: Adverse events.

	Safety Population	
	Oral NaCl Hydration (n = 134)	Intravenous Hydration (n = 137)
Total adverse events	31 (23.1%)	6 (4.4%)
Total adverse events related to study treatment	27 (20.1%)	2 (1.5%)
Nausea	13 (9.7%) *	0
Vomiting	10 (7.5%) *	0
Abdominal discomfort	3 (2.2%) *	0
Dyspnea	0	2 (1.5%) †
Malaise/asthenia	1 (0.7%) *	1 (0.7%) *
Headache	1 (0.7%)	0
Leg cramps	1 (0.7%)	0
Pruritus	1 (0.7%)	0
Allergic reaction to contrast	1 (0.7%)	0
Bronchospasm	0	1 (0.7%) ‡
Chest tightness	0	1 (0.7%)
Infection (bronchopneumonia and cystitis)	0	1 (0.7%) ‡

Data are expressed as n (%). * Adverse events related to study treatment according to the investigator criteria (adverse reactions). † One of the two dyspnea cases was related to study treatment (adverse reaction). ‡ Serious adverse events requiring hospitalization, not related to study treatment.

## Data Availability

The data presented in this study are available on request from the corresponding author. The data are not publicly available due to participant confidentiality.

## Appendix A

**Table A1:** Patient characteristics at inclusion in the study (intention-to-treat population).

	Intention-to-Treat Population	
	Oral NaCl Hydration (n = 134)	Intravenous Hydration (n = 137)
Age, years	74.2 [6.2]	74.5 [6.7]
Sex, male	96 (71.6%)	84 (61.3%)
Hypertension	102 (76.1%)	103 (75.2%)
Diabetes	95 (70.9%)	103 (75.2%)
Smoking		
Ex-smoker	79 (59.0%)	69 (50.4%)
Current smoker	7 (5.2%)	10 (7.3%)
Cancer	76 (56.7%)	63 (46.0%)
Chronic kidney disease ^a,^	60 (44.8%)	52 (38.0%)
Heart failure	19 (14.2%)	27 (19.7%)
Peripheral artery disease	11 (8.2%)	8 (5.8%)
eGFR, mL/min/1.73 m^2^	65.7 [19.5]	68.8 [19.5]
Serum creatinine, mg/dL	1.10 [0.32]	1.02 [0.29]
Cystatin C, mg/dL	1.37 [0.39]	1.41 [0.46]
Albumin-to-creatinine ratio, mg/g	17.4 [71.8]	19.5 [48.5]
Urea, mg/dL	49.7 [17.0]	46.6 [18.6]
Serum sodium, mg/dL	139.3 [2.6]	139.7 [3.1]
Serum potassium, mg/dL	4.5 [0.4]	4.5 [0.5]
HbA1c, %	6.6 [1.0]	6.7 [1.5]
BNP, pg/mg	56.6 [126.6]	54.8 [70.7]
Non-diuretic antihypertensives	101 (75.4%)	102 (74.5%)
Non-insulin antidiabetic drugs	87 (64.9%)	91 (66.4%)
Lipid-lowering agents	83 (61.9%)	85 (62.0%)
Diuretics	51 (38.1%)	50 (36.5%)

Patient characteristics collected at inclusion, before the CE-CT scan, prior to study treatment exposure. Data are expressed as n (%), median [interquartile range]. ^a^ Chronic kidney disease defined as eGFR (MDRD-4) < 60 mL/min/1.73 m^2^. Abbreviations: eGFR: estimated glomerular filtration rate according to MDRD-4; BNP: brain natriuretic peptide.

**Table A2:** Subgroup analysis (per-protocol population).

	Per-Protocol Population		
	Oral NaCl Hydration Events/N	Intravenous Hydration Events/N	Risk Difference (95% CI) *
**Age**			
≥80 years	3/23 (13.0%)	2/30 (6.7%)	6.4% (−13.0%; 28.7%)
66 to 79 years	2/100 (2.0%)	2/99 (2.0%)	0.0% (−6.0%; 6.0%)
**Diabetes**			
Yes	4/87 (4.6%)	4/97 (4.1%)	0.5% (−6.9%; 8.4%)
No	1/36 (2.8%)	0/32 (0.0%)	2.8% (−10.8%; 16.2%)
**Heart failure**			
Yes	2/16 (12.5%)	2/25 (8.0%)	4.5% (−17.6%; 32.4%)
No	3/107 (2.8%)	2/104 (1.9%)	0.9% (−5.0%; 6.9%)
**Chronic kidney disease**			
30 to 59 mL/min/1.73 m^2^	2/53 (3.8%)	1/47 (2.1%)	1.7% (−9.4%; 12.2%)
≥60 mL/min/1.73 m^2^	3/70 (4.3%)	3/82 (3.7%)	0.6% (−7.4%; 9.6%)

* Non-inferiority of oral prophylaxis would be demonstrated if the upper limit of the 95% CI of the absolute risk difference between groups was less than 5% (non-inferiority margin). The 95% confidence interval for the difference between two independent proportions was calculated according to Wilson’s method with continuity correction.

**Table A3:** Differences in continuous efficacy assessments between oral- and intravenous-hydration groups within the 48-h follow-up period in the intention-to-treat population.

Variable	Intention-to-Treat Population		
	Oral NaCl Hydration (n = 134)	Intravenous Hydration (n = 137)	*p*
eGFR, mL/min/1.73 m^2^			
24 h	66.2 ± 19.1	68.6 ± 18.7	0.298
48 h	65.7 ± 19.9	67.5 ± 19.5	0.476
Serum creatinine, mg/dL			
24 h	1.10 ± 0.33	1.02 ± 0.28	0.027
48 h	1.11 ± 0.36	1.04 ± 0.31	0.115
Cystatin C, mg/dL			
24 h	1.39 ± 0.44	1.34 ± 0.47	0.441
48 h	1.37 ± 0.44	1.37 ± 0.46	0.918
Albumin-to-creatinine ratio, mg/g			
24 h	17.9 [56.4]	17.6 [53.9]	0.656
48 h	17.4 [60.2]	18.9 [40.7]	0.819
Urea, mg/dL			
24 h	43.8 ± 18.5	42.3 ± 18.0	0.546
48 h	45.8 ± 19.2	43.4 ± 19.9	0.360
Serum sodium, mg/dL			
24 h	139.5 ± 2.6	139.3 ± 2.6	0.627
48 h	139.2 ± 2.7	139.5 ± 2.6	0.351
Serum potassium, mg/dL			
24 h	4.4 ± 0.4	4.4 ± 0.5	0.917
48 h	4.4 ± 0.4	4.5 ± 0.5	0.528

Data expressed as mean ± standard deviation or median [interquartile range]. eGFR: estimated glomerular filtration rate according to MDRD-4.
